# Dyslipidemia Might Be Associated with an Increased Risk of Osteoarthritis

**DOI:** 10.1155/2020/3105248

**Published:** 2020-02-15

**Authors:** Jianping Xiong, Junyu Long, Xi Chen, Ye Li, Hai Song

**Affiliations:** ^1^Department of Surgery, Peking Union Medical College Hospital, Chinese Academy of Medical Sciences and Peking Union Medical College (CAMS & PUMC), Beijing 100730, China; ^2^Department of Orthopedics, Peking Union Medical College Hospital, Chinese Academy of Medical Sciences and Peking Union Medical College (CAMS & PUMC), Beijing 100730, China; ^3^Department of Science and Education, Tangshan People's Hospital, Tangshan 063000, China

## Abstract

**Background:**

According to several studies, the autoimmune response may lead to osteoarthritis and dyslipidemia and may affect the homeostasis of the human body's internal environment and then cause its own immune regulation. Consequently, the risk of osteoarthritis might be increased by dyslipidemia, but this association is not universally acknowledged. Therefore, a systematic review and meta-analysis was conducted to study the relationship between dyslipidemia and the risk of osteoarthritis.

**Methods:**

In this study, PubMed, EMBASE, and the ISI Web of Science were used to identify related studies published before July 2018. The relationship between dyslipidemia and the risk of osteoarthritis was evaluated on the basis of relative risk (RR) values and the corresponding 95% confidence intervals (CIs). To further investigate this relationship, we also employed the random effects model proposed by DerSimonian and Laird.

**Results:**

A total of nine studies were included to study the effect of dyslipidemia on the risk of osteoarthritis, including four cohort, three case-control, and two cross-sectional studies. Among these studies, six stated data for knee osteoarthritis, two reported on hand osteoarthritis, and one reported on hip osteoarthritis. A total of 53,955 participants were included in the meta-analysis, comprising 22,501 patients with OA (19,733 hand OA, 2,679 knee OA, and 89 hip OA). Based on the meta-analysis of case-control and cross-sectional studies, osteoarthritis was clearly higher in those with dyslipidemia compared to those who did not suffer from dyslipidemia (case-control: OR = 1.37; 95%CI = 1.27–1.46; cross-sectional: OR = 1.33; 95%CI = 1.21-1.46). In addition, the meta-analysis of cohort studies did not present any relationship between dyslipidemia and OA (RR = 1.00; 95%CI = 0.85–1.14).

**Conclusions:**

Even though our meta-analysis of case-control and cross-sectional studies suggested a strong relationship between dyslipidemia and osteoarthritis; this relationship was not validated by our meta-analysis of only cohort studies. As a result, further investigation needs to be conducted on the relationship between dyslipidemia and osteoarthritis, considering the significant public health relevance of the topic.

## 1. Introduction

Osteoarthritis (OA) refers to a chronic degenerative disease that involves the cartilage, as well as its surrounding tissues [1]. OA is considered the most common joint disease, and nearly 10-12% of the population suffers from OA [2]. In addition, it is expected that this number will increase dramatically due to the quickly increasing aging population combined with the growing prevalence of obesity [3]. Consequently, osteoarthritis is considered to have a negative influence on the health economy [4]. It can be forecast that by the year 2032, an additional 26,000 per million patients over the age of 45 will present to their general practitioner with osteoarthritis compared to 2012 [5]. OA is associated with age, female gender, obesity, joint injury, and career, as well as a high level of physical activity [5]. In addition, the autoimmune response of the synovium plays an important role in rheumatoid arthritis. In recent years, the immunological pathogenesis of synovium in osteoarthritis has attracted the attention of many researchers. Whether the immune mechanism and inflammatory mediators are involved in the occurrence and development of osteoarthritis deserves further discussion. This may provide a new research idea for the pathogenesis of osteoarthritis, to improve our understanding of the development of this disease and change the way of treatment. In recent years, research has shown that metabolic syndrome is closely associated with OA, which is even a part of generalized metabolic disorder. Metabolic syndrome is composed of a bundle of interrelated metabolic risk factors, including diabetes, obesity, dyslipoproteinemia, and hypertension [6]. Furthermore, the incidence of metabolic syndrome is very high; it has been estimated to be as high as 26.7% in industrialized countries [6]. In the context of musculoskeletal disorders, metabolic syndrome has increasingly gained more attention because of its relationship with knee OA [7]. Obesity, the main feature in metabolic syndrome, is overwhelmingly related to degenerative joint changes in regard to mechanical load [8]. Alternatively, obesity-related OA can afflict nonweight-bearing joints (e.g., the hands), signifying a role of adipokines (circulating mediators released by adipose tissue), such as leptin. Thus, OA may have a systemic metabolic element [9]. In addition, OA can be categorized into three phenotypes: metabolic OA, age-related OA, and injury-related OA [10]. Nevertheless, as one of the components of metabolic syndrome, the role of dyslipidemia in the pathogenesis of OA is not completely understood. Dyslipidemia may affect the homeostasis of the human body's internal environment and then cause its own immune regulation. Dyslipidemia is related to chronic low-grade inflammation and oxidative stress, likely increasing the development of OA [11, 12]. A survey carried out by Ghandehari concluded that approximately 51.4 million US adults presented with high cholesterol and triglycerides, in addition to 36.1 million with elevated low-density lipoproteins [13]. As a result, we chose to conduct a systematic review and meta-analysis of the published observational studies to better comprehend the relationship between dyslipidemia and the risk of OA.

## 2. Materials and Methods

This research was performed according to the Preferred Reporting Items for Systematic Reviews and Meta-Analyses (PRISMA) Statement [14] and the Meta-analysis of Observational Studies in Epidemiology (MOOSE) guidelines [15].

### 2.1. Data Sources and Search Strategy

Published studies in PubMed, EMBASE, and the Web of Science were searched based on the following keywords: (“hyperlipidaemia” OR “dyslipidemia” OR “triglyceride” OR “cholesterol” OR “lipoprotein” OR “lipid” OR “metabolic syndrome”) and (“OA”). No restrictions on language or the date of publication were placed. Additionally, this study also searched the reference lists. Unpublished studies and original data were not included.

### 2.2. Eligibility Criteria for Study Selection

The eligibility criteria were as follows: study design (randomized controlled trials and cohort, case-control, or cross-sectional studies); an exposure factor of blood lipid levels and an outcome of OA; availability of the odds ratio (OR)/risk ratio (RR) values and corresponding 95% confidence intervals (CIs) for dyslipidemia patients and the general population; or the availability of sufficient information to measure these variables. The most recent all-inclusive study was searched under the condition that two studies used the same population. The definition of dyslipidemia was in line with the US National Cholesterol Education Program Adult Treatment Panel III guidelines. In accordance with the National Cholesterol Education Program, the definition of dyslipidemia was high-density lipoprotein cholesterol (HDL‐C) < 40 mg/dL, as well as total cholesterol, low-density lipoprotein cholesterol (LDL-C), and TG levels of ≥200, ≥130, and ≥130 mg/dL, respectively [5]. The definition of osteoarthritis was in line with the American College of Rheumatology (ACR) clinical and clinical plus radiographic criteria [6]. The ACR classification criteria for (OA) permits the categorization of individuals for hand, knee, and hip OA [6]. We strictly abided by this classification standard.

### 2.3. Data Abstraction and Quality Assessment

Two scholars (J.X. and J.L.) obtained the essential information from the chosen studies according to the standard. The following information was gathered: name of the first author, publication year, country in which the research was carried out, study design, number of participants, period of follow-up, sources of controls, potential adjusted confounding variables, OR/RR values, and 95% CIs.

To date, no available common scale has been proposed to evaluate the quality of all kinds of observational studies. As a result, two authors individually employ the modified Newcastle-Ottawa Scale (NOS) [16] as reported by Zhu et al. [17] to assess the quality of the included studies. Quality types were allocated in accordance with the scores of each study, consisting of high quality (score 7-9), medium quality (score 4-6), and low quality (score less than 4) [18]. The maximum total score could reach 9 points, and discrepancies were solved by mutual agreement.

### 2.4. Statistical Analysis

The random effects model put forward by DerSimonian and Laird was applied to investigate the relationship between dyslipidemia and the risk of OA among the cohort studies [19]. The *I*^2^ statistic was employed to evaluate heterogeneity between the studies. Low, medium, and high heterogeneities were categorized as 25%, 50%, and 75%, respectively [20]. Definite heterogeneity was assumed if the *p* value was less than 0.1. Sensitivity analyses were conducted by altering the pooling model [21]. In addition, a sensitivity analysis was carried out to evaluate the influence of each individual study on the summarized estimate by means of successively excluding one research study at a time. Publication bias was assessed using Begg's [22] and Egger's [23] tests. No testing for funnel plot asymmetry was carried out due to the limited number of studies included in the analysis (*n* < 10) [24].

Furthermore, we conducted a meta-analysis of the case-control and cross-sectional studies in regard to the influence of dyslipidemia on the risk of OA and expressed the results as pooled risk ratios with 95% CIs with the application of a random effects model.

STATA version 12.0 (Stata) was carried out to perform all statistical analyses.

## 3. Results

### 3.1. Study Selection and Study Characteristics

**Figure 1:**
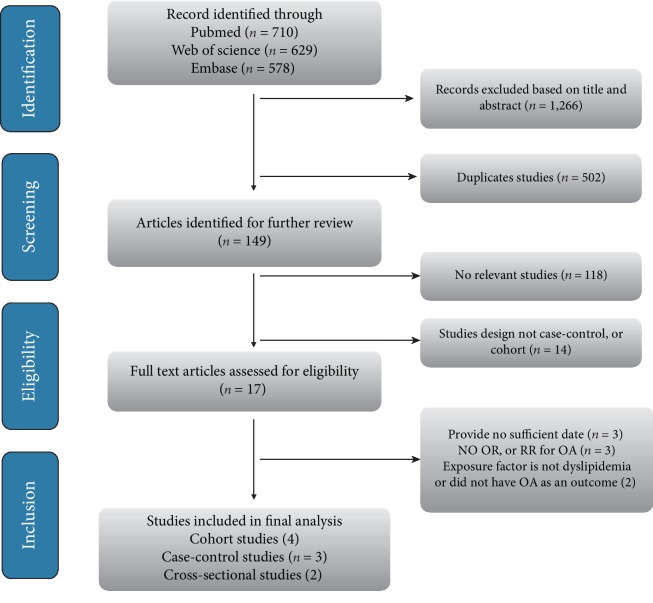
Study selection process for the meta-analysis.

The process of study selection for the meta-analysis can be found in Figure 1. In total, 1,917 articles were obtained through the initial search, and 502 were duplicates. An additional 1,266 studies were removed based on the title and abstract. Eventually, after evaluation of the full texts, eight studies were excluded for the reason that they did not satisfy our inclusion criteria: three studies offered inadequate information [25–27], three studies did not offer ORs or RRs for OA or adequate information to calculate these variables [28–30], and two studies were removed that either did not have dyslipidemia as an exposure or did not have OA as an outcome [31, 32]. Ultimately, nine available observational articles were recognized for our meta-analysis [33–41].

Table 1 presents the principal features of the studies included in the meta-analysis, all of which were observational studies. The studies were conducted in the following countries: two in China, one in the UK, one in Germany, one in Switzerland, one in Sweden, one in Australia, one in Japan, and one in Korea. Four studies were cohort studies, three were case-control studies, and two were cross-sectional studies. All the studies included the levels of serum TC and TGs, as well as LDL and HDL cholesterol as exposures. A total of 53,955 participants were included in the meta-analysis, comprising 22,754 patients with OA (19,733 hand OA, 2,679 knee OA, and 89 hip OA). The mean age of patients ranged from 46.5 to 81.2 years, and the presence of hypertension and diabetes mellitus was described by 17.5% to 79.3% and 7.1% to 48.5% of patients, respectively. Data from 1988 to 2014 were collected. The follow-up period was within the range of 1 to 13 years. The modified NOS scores for the included studies ranged from 5 to 9, including seven high-quality studies and two medium-quality studies (Table 2). A large proportion of the studies offered risk estimates that were adjusted for age (6 studies), smoking (6 studies), gender (4 studies), physical activity (4 studies), and alcohol consumption (3 studies). Fewer studies were adjusted for lipid-lowering agents/statins (2 studies) and BMI (1 study) (Table 1).

### 3.2. Cohort Studies

**Figure 2:**
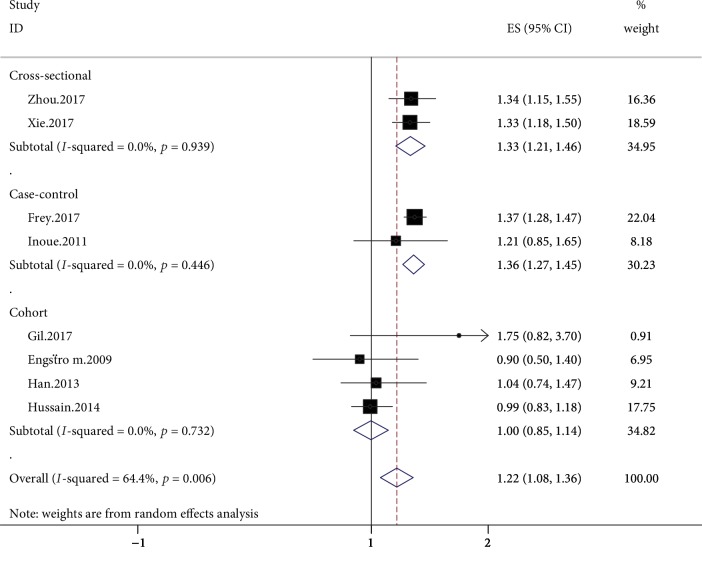
Forrest plot showing the relationship between dyslipidemia and the risk of OA, using a random effects model and depicted on a logarithmic scale. Squares represent the risk estimates for each individual study. Horizontal lines represent the 95% confidence intervals, and diamonds represent the summary risk estimates with 95% confidence intervals. CI: confidence interval; ES: effect size.

**Figure 3:**
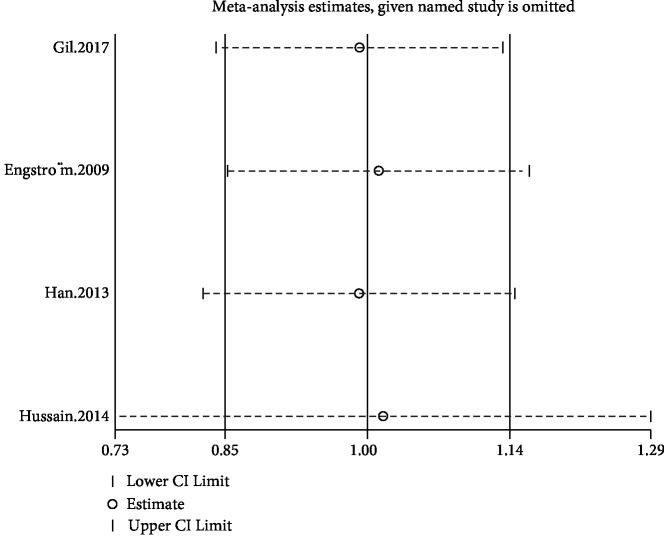
Sensitivity analysis of the association between dyslipidemia and the risk of OA (cohort study).

Reports from four studies permitted the calculation of effect estimates for OA [34–36, 38]. All of the cohort studies were population based, and the follow-up was in the range of 1 to 3 years. Among them, two studies reported data for knee OA, one study reported on hand OA, and one study reported on hip and knee OA. In the pooled analysis, dyslipidemia exerted a null influence on the risk of OA (RR = 1.00; 95%CI = 0.85–1.14; *I*^2^ = 0%) (Figure 2). In the sensitivity analysis, the general results for the relationship between dyslipidemia and OA kept steady with the changes of the pooling model (fixed: RR = 1.00; 95%CI = 0.85–1.14). In addition, when we successively removed each study in turn to evaluate the stability of the results, we found that no study likely influenced the pooled risk estimate (Figure 3). No test for funnel plot asymmetry was carried out due to the limited number of included studies (*n* < 10). Nevertheless, Begg's (*p* = 0.697) and Egger's (*p* = 0.465) tests failed to identify substantial publication bias.

### 3.3. Case-Control Studies

The relationship between dyslipidemia and the risk of OA was investigated by three case-control studies [33, 40, 41]. Two case-control studies were population-based, and one case-control study was hospital based. According to Frey et al. [33] and Sturmer et al. [41], there exists an increase in the risk of knee OA among dyslipidemia patients. Nevertheless, the results obtained by Inoue et al. [40] suggested that dyslipidemia does not obviously change the risk of hand OA. There exist significant pooled estimates of the effect of cirrhosis without any considerable heterogeneity (OR = 1.37; 95%CI = 1.27–1.46; *I*^2^ = 0%) (Figure 2). Based on the general results, the risk of OA was obviously higher among dyslipidemia patients in comparison with the general population. In accordance with the sensitivity analysis, despite excluding studies where the sources of controls were not hospital-based, the relationship between dyslipidemia and OA remained steady. In addition, the general results regarding the relationship between dyslipidemia and OA were kept under the condition that the pooling model was changed (fixed effects model: OR = 1.37; 95%CI = 1.27–1.46; random effects model: OR = 1.37; 95%CI = 1.27–1.46). Finally, when we successively removed each study in turn to evaluate the stability of the results, no research likely influenced the pooled risk estimate.

### 3.4. Cross-Sectional

Two hospital-based, cross-sectional studies reported the influence of dyslipidemia on the risk of OA [37, 39]. Both studies reported data on knee OA, and both reported an obvious connection of dyslipidemia with OA. The random effects meta-analysis also demonstrated that dyslipidemia considerably increased the risk of OA, without heterogeneity (OR = 1.33; 95%CI = 1.21-1.46; *I*^2^ = 0%) (Figure 2).

## 4. Discussion

Admittedly, this is the first meta-analysis to investigate the relationship between dyslipidemia and the risk of OA. Even though a meta-analysis of cohort studies did not present any connection between dyslipidemia and OA, a meta-analysis of case-control and cross-sectional studies demonstrated that the risk of OA was obviously higher among those suffering from dyslipidemia compared to those without dyslipidemia.

The potential pathophysiological mechanisms accounting for these results remain unknown. Changed expression of cholesterol influx genes in human osteoarthritic chondrocytes and in the cartilage of patients with OA has been depicted [42]. Moreover, in vivo studies have demonstrated that decreased high-density lipoprotein cholesterol plays an important role in the pathogenesis of OA. Based on early research, dietary cholesterol intake has been shown to increase spontaneous cartilage damage in mice [43]. Correspondingly, high LDL levels stimulate synovial inflammation, as well as ectopic bone formation, in mouse OA models [43]. LDL could be involved in the development and progression of OA through the stimulation of synovial cells and chondrocytes [44].

There are several strengths of our study. First, this is the first meta-analysis to investigate the relationship between dyslipidemia and the risk of OA with a large sample size (22,754 cases of OA and 53,955 participants), probably providing a reference in regard to this relationship. Clinically, the results indicate that patients with dyslipidemia are required to focus more on their risk of OA in comparison with the general population. Second, a comprehensive literature search was conducted using PubMed, EMBASE, and the Web of Science to recognize potential studies from which to examine the relationship between dyslipidemia and the risk of OA. Additionally, most of the studies included were high-quality studies. All of these features enhance the reliability of our study.

However, this study still has some limitations. First, a meta-analysis of the different locations of OA, such as the knee, hip, and hand, was not conducted due to the limited number of included studies. The effect of dyslipidemia on the risk of knee vs. hip OA may have differences because of the diverse susceptibility of these two joints to metabolic factors. The knee may have more dependence on soft tissue and neuromuscular control for its stability. Comparatively, in the hip, the bony shape and joint congruence seem to have greater significance on the development of hip OA, which makes the hip less vulnerable to the effects of inflammation. Second, the observed outcome was an association, which is considered a confounding bias. Seven studies reported data on knee or hip osteoarthritis, but not all of the studies were adjusted for BMI or physical activity. Zhou et al. [39], Xie et al. [37], Engstrom et al. [35], Han et al. [36], and Monira Hussain et al. [34] reported on knee or hip osteoarthritis adjusted for physical activity (activity level). Hussain et al. [34] reported on knee osteoarthritis adjusted for BMI. Two studies (Inoue et al. [40] and Sturmer et al. [41]) that reported data on knee osteoarthritis did not adjust for BMI or physical activity. There were few data on knee OA patients compared with normal participants, because the studies by Inoue et al. [40] and Sturmer et al. [41] are aimed at investigating the data of the general population. Even though most studies were adjusted for recognized risk factors for OA, such as age, alcohol consumption, and smoking, many potential adjustment factors remained unclear, such as cardiovascular disease and obesity, showing a close association with the development of OA. Only two studies in the meta-analysis adjusted the OR/RR with lipid-lowering agents/statins. Additionally, we did not succeed in obtaining information concerning medication use, especially regarding lipid-lowering agents such as statins, which could have an influence on the development of OA. It has been indicated that statins might play a protective role in developing OA, likely caused by pleiotropic anti-inflammatory properties or by enhancing chondrogenesis [45, 46]. Third, a meta-analysis of the effects of various kinds of dyslipidemia, such as low-density lipoprotein levels, triglyceride levels, and cholesterol levels, was not carried out due to the limited number of included studies. Ultimately, a large proportion of the included studies were cohort studies, yet we also included two cross-sectional studies and three case-control studies. Case-control studies tend to have recall and selection biases, and cross-sectional studies are insufficient for assessing the relationship between cirrhosis and the risk of stroke. It must also be admitted that a meta-analysis cannot deal with the limitations of the included studies. Finally, it is difficult to determine the causality of this relationship on the basis of these observational studies alone given the heterogeneity of the results and the limited data from prospective studies, which were restricted to 4 studies that, respectively, included 89, 270, 143, and 660 cases of OA, as well as the lack of information about time-related factors. Although we were not always able to ascertain the source of the heterogeneity, we performed several sensitivity and subgroup analyses to address this issue.

In conclusion, considering there was a lack of evidence from the cohort studies but that strong connections were noted in the case-control and cross-sectional studies, the current systematic review and meta-analysis offers inadequate but fascinating evidence of the effects of dyslipidemia on the risk of OA. In general, the findings indicate that dyslipidemia might exert a significant pathogenic role in the development of OA, as well as offers a rationale for the shared care of patients by metabolic physicians. To be specific, structured clinical trials with predefined criteria for patient selection are still required to investigate the role of lipid-lowering therapies in the management of OA.

## Figures and Tables

**Table 1:** Main characteristics of the included studies. RR, relative risk; OR, odds ratio; CI, confidence interval; BMI: body mass index; CHD, coronary heart disease; COPD, chronic obstructive pulmonary disease; NR: not reported.

Author/year of publication	Country	No. of case/control	Follow-up period	Source of controls	Site of OA	Study subtype	Adjusted factors	Adjusted OR/RR (95% CI)
Zhou/2017	China	281/3066	2008-2013	Hospital	Knee	Cross-sectional	Age (as a continuous variable), WHR (as a continuous variable), gender, physical workload, physical exercise, smoking, and drinking	1.34 (1.15–1.55)
Frey/2017	Switzerland	19,590/19,590	1995-2014	Population	Hand	Case-control	Smoking, alcohol consumption, diabetes mellitus, hypertension, COPD, hand fractures, hormone replacement therapy, osteoporosis, and statin use	1.37 (1.28-1.47)
Xie/2017	China	1669/4095	2013-2014	Hospital	Knee	Cross-sectional	Age, gender, activity level, smoking status, alcohol drinking status, and educational background	1.33 (1.18–1.50)
Gil/2017	UK	143/707	1988-1989	Population	Hand	Cohort	Age, any current medication, diabetes medication, statin use, hormone replacement therapy (HRT), previous CVD, menopause, smoking, body mass index (BMI), and systolic and diastolic blood pressure	1.75 (0.82–3.70)
Engstrom/2009	Sweden	89/5082	1991-1994	Population	Knee and hip	Cohort	Age, gender, smoking, physical activity, and CRP	0.9 (0.5-1.4)
Han/2013	Korea	270/1964	2008-2009	Population	Knee	Cohort	Age, height, exercise, alcohol intake, and smoking	1.04 (0.74, 1.47)
Inoue/2011	Japan	52/243	1995-2005	Population	Knee	Case-control	NR	1.21 (0.85-1.65)
Hussain/2014	Australia	660/19,208	2003-2007	Population	Knee	Cohort	Age, gender, country of birth, level of education, physical activity and BMI	0.99 (0.83–1.18)
Sturmer/1998	Germany	809/809	NR	Hospital	Knee	Case-control	NR	1.61 (1.06-2.47)

**Table 2:** Modified Newcastle-Ottawa Scale scores for the included studies. The asterisks represent a score (number of stars).

Author/year of publication	Fully defined cases	Defines the study design	Selection of controls	Describes the general characteristics	Controlled for important factors or confounding factors	Lists of inclusion and exclusion criteria for all participants	Provides enrollment duration for all participants	Indicates study period and follow-up duration	Total score
Zhou/2017	^∗^	^∗^	^∗^	^∗^	^∗∗^	^∗^	^∗^	^∗^	9
Frey/2017	^∗^	^∗^		^∗^	^∗∗^	^∗^	^∗^	^∗^	8
Xie/2017	^∗^	^∗^	^∗^	^∗^	^∗∗^	^∗^	^∗^		8
Gil/2017	^∗^		^∗^	^∗^	^∗∗^	^∗^		^∗^	7
Engstrom/2009	^∗^	^∗^		^∗^	^∗^	^∗^	^∗^	^∗^	7
Han/2013	^∗^	^∗^	^∗^	^∗^	^∗^	^∗^	^∗^	^∗^	8
Inoue/2011	^∗^	^∗^		^∗^			^∗^	^∗^	5
Hussain/2014	^∗^	^∗^	^∗^		^∗^	^∗^	^∗^	^∗^	7
Sturmer/1998	^∗^	^∗^	^∗^			^∗^	^∗^		5
